# Role of mitochondrial complex I genes in host plant expansion of *Bactrocera tau* (Tephritidae: Diptera) by CRISPR/Cas9 system

**DOI:** 10.1111/1744-7917.13495

**Published:** 2025-01-19

**Authors:** Wei Shi, Linsheng He, Ruixiang Li, Jun Cao

**Affiliations:** ^1^ Ministry of Education Key Laboratory for Transboundary Ecosecurity of Southwest China, Yunnan Key Laboratory of Plant Reproductive Adaptation and Evolutionary Ecology, School of Ecology and Environmental Science, Institute of Biodiversity Yunnan University Kunming China; ^2^ School of Life Science Yunnan University Kunming China

**Keywords:** *Bactrocera tau*, gene knockout, host expansion, invasive pests, mitochondrial complex I gene, tephritids

## Abstract

Host expansion facilitates tephritid flies to expand their ranges. Unraveling the mechanisms of host expansion will help to efficiently control these pests. Our previous works showed mitochondrial complex I genes *Ndufs1*, *Ndufs3*, and *Ndufa7* being upregulated during host expansion of *Bactrocera tau* (Walker), one of the highly hazardous species of tephritids. However, their roles in the host expansion of *B. tau* remain unknown. Here, using clustered regularly interspaced short palindromic repeats (CRISPR) / CRISPR‐associated nuclease 9 (Cas9) editing system for the first time, a stable homozygous *Ndufa7* strain (*Btndufa7^−/−^*), heterozygous *Ndufs1* (*Btndufs1^+/−^*), and *Ndufs3* strains (*Btndufs3^+/−^*) were obtained from F3 generation of *B. tau*, after gene knockout. Reduced sizes of larvae and pupae of the *Ndufa7* knockout strain were first observed. Notably, the mean values of fitness estimation (pupal numbers, single‐pupal weight and emergence rate) and *Ndufa7* gene expression in the *Ndufa7* knockout strain were slightly reduced on 2 native hosts (summer squash and cucumber), while it sharply decreased on the novel host banana and the potential host pitaya, compared with those of the wild‐type strain. Furthermore, the *Ndufa7* knockout strain did not survive on the novel host guava. These results suggested that *Ndufa7* disturbs the survival on native hosts, expansion to novel hosts, and further expansion to potential hosts of *B. tau*. Homozygous lethality occurred after the knockout of *Ndufs1* or *Ndufs3*, suggesting that these 2 genes play a role in the early development of *B. tau*. This study revealed that *Ndufa7* is a target gene for the management of tephritids and opens a new avenue for pest control research.

## Graphical abstract

The role of 3 mitochondrial complex I gene in host expansion of *Bactrocera tau* was verified by CRISPR/Cas9 gene editing system. A stable homogenous strain (*Btndufa*7‐/‐) of *B.tau* was established after silencing *Ndufa7*. The reduced size of larvae and pupae of *Ndufa7* knockout strain was first found.Then different levels of fitness and relative expression of *Ndufa7* gene of *B.tau* on various host plants were found in *Ndufa7* knockout strain. These results shown that *Ndufa7* has a role in disturbing native hosts survival, novel hosts and potential host expansion of *B.tau*. Heterozygous *B.tau* strains *Btndufs*1+/‐ and *Btndufs*3+/‐ were obtained respectively in F3 generation and homozygous lethality occurred after knockout *Ndufs1* and *Ndufs3* genes. This result suggested that the two genes respectively had a role in tephritids early development.

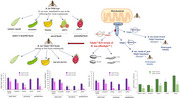

## Introduction

Host expansion refers to herbivores’ abilities to use novel hosts but not lose their abilities to use native hosts (Piñero *et al.*, 2017). This phenomenon is common among phytophagous insects, including highly hazardous pests. Host expansion facilitates the spread of pests and causes severe damage (Charlery de la Masselière *et al.*, 2017). Therefore, revealing the mechanisms of host expansion in herbivorous pests can help develop efficient ways to manage these pests.

Tephritid flies (Diptera) are invasive herbivorous pests that damage fruit and vegetables (Garcia *et al.*, 2020). Host expansion is typical in tephritids such as *Zeugodacus cucurbitae* (Coquillett) (Vayssières *et al.*, 2007), *Rhagoletis pomonella* (Walsh), and *Bactrocera tau* (Walker) (Shi *et al.*, 2020a). *B. tau*, which mainly attacks hosts of the family Cucurbitaceae, is a highly hazardous species (Christenson & Foote, 1960). Recently, *B. tau* has expanded from native Cucurbitaceae hosts to other hosts across different genera (Sumrandee *et al.*, 2011), such as Leguminosae (e.g., *Phaseolus vulgaris* L.) (Sumrandee *et al.*, 2011), Myrtaceae (e.g., *Psidium guajava* L.) (Hasyim *et al.*, 2008), and Sapotaceae (e.g., *Manilkara zapota* L.) (Huang *et al.*, 2005). Overall, the host range of *B. tau* has expanded to include more than 80 species (Huang *et al.*, 2005). Many of these novel hosts to which *B. tau* successfully expands are plants of tropical and sub‐tropical species (Zhang & Chen, 2018). Currently, *B. tau* is expanding northward in China. This species has been reported in Shanxi and Gansu (Wang *et al.*, 1994; Yang *et al.*, 1994), two northern Chinese provinces. The risk of *B. tau* expanding to other hosts, including temperate hosts, is increasing.

Tephritids must adapt to volatile organic compounds (VOCs) and other secondary metabolites in the novel host fruits to successfully expand and use a new host (Shi *et al.*, 2022b). Different chemical stressors in different hosts drive multifaceted adaptations in flies, including behavioral, physical, and even neurological adaptations (Tallamy, 2000). Regardless of the adaptation type, gene activity is strongly involved in regulating these processes. Thus, exploring which genes contribute to the host expansion of tephritids is important for the development of new control technologies, such as RNA interference (RNAi) methods.

Taking *B. tau* as an example, high‐throughput sequencing has been used to search for genes related to host expansion in our previous works (Shi *et al.*, 2020a). The subunits of mitochondrial complex I (CI), namely, *Ndufs1*, *Ndufs3*, and *Ndufa7* (Su *et al.*, 2016; Xiao *et al.*, 2022) of *B. tau* feeding on the novel host banana were upregulated, but not feeding on native Cucurbitaceae hosts (Shi *et al.*, 2020a). Moreover, the associated oxidative phosphorylation (OXPHOS) pathways *of B. tau* feeding on novel host bananas were highly enriched (Shi *et al.*, 2020a). The OXPHOS pathway is mainly responsible for energy metabolism, which begins with mitochondrial CI (Ali & Dholaniya, 2022). Enrichment of the OXPHOS pathway further suggests that CI genes are involved in this process. Therefore, we hypothesized mitochondrial CI genes probably play roles in the host expansion of *B. tau*. Their roles in host expansion need to be further verified.

Host expansion of tephritids is a series of complex biological processes involving development, detoxification, digestion and so forth, which require a supply of energy. Here, we speculated that mitochondrial CI genes may be closely associated with the host expansion of invasive insects. In the present study, we focused on 3 mitochondrial CI genes (*Ndufa7*, *Ndufs1*, and *Ndufs3*) and examined whether they affected the host expansion ability of the invasive insect *B. tau*.

## Materials and methods

### Insects

Pumpkin (*Cucurbita moschata*) fruits damaged by *B. tau* were brought to the Yunnan University Laboratory in Yuanjiang County, Yuxi, Yunnan Province. These fruits were placed in a sandbox in a cage (30 cm × 30 cm × 60 cm) to hatch into pupae. After the pupae emerged into adults, only *B. tau* flies were continuously screened and reared. *B. tau* flies were fed fresh pumpkin slices to enlarge the population for subsequent experiments and were reared in a room at 25 ± 1 °C and 80% relative humidity.

### Knockout of the 3 CI genes

**Knockout gene selection and polymerase chain reaction (PCR)** We targeted 3 mitochondrial CI genes, *Ndufs1*, *Ndufs3*, and *Ndufa7*, for knockout. Total RNA was extracted from an adult *B. tau* individual using TRIzol reagent (Invitrogen, CA, USA). Then, 1 *µ*g of total RNA was reverse transcribed into complementary DNA (cdna) using a PrimeScript RT reagent Kit (Takara, Dalian, China). The protein‐coding regions of the 3 CI genes were amplified separately from the cdna templates using their respective primers (Table 1A). These primers were designed based on the corresponding sequences from *Z. cucurbitae* (LOC105212758, LOC105211095, and LOC105208622), a species related to *B. tau*. The PCR amplification was conducted using 2 × Tag PCR MasterMix (TIANGEN, Beijing, China) and a 3‐step generic PCR cycle: 95°C for 3 min, followed by 20 cycles of 95°C for 30 s, 65°C for 30 s, and 72°C for 1 min; then by 15 cycles of 95°C for 30 s, 55°C for 30 s, 72°C for 1 min.

**Table 1:** Primers and sequences

(A) Primers used for polymerase chain reaction amplification of the 3 target genes.	
Primers (target sequencing)	Sequences
*Ndufa7*‐ID‐S (5′–3′)	GTGATGTTGCGACACTTCTTC
*Ndufa7*‐ID‐A (3′–5′)	CGCCAGGTGTTGGTAACTT
*Ndufs1*‐ID‐S (5′–3′)	AATTGTCGCATGTGCCTTGTA
*Ndufs1*‐ID‐A (3′–5′)	CGCCATAGCCTGATCTTGAAG
*Ndufs3*‐ID‐S (5′–3′)	ATATGTAGCCGAATGCCTTC
*Ndufs3*‐ID‐A (3′–5′)	GACTTCATTACACGAATCCAG

(B) Single guide RNA (sgRNA) target sequences and sgRNA sequences for the 3 complex I (CI) genes.		
Genes	Target sequences (5ʹ–3ʹ)	sgRNA sequences (5ʹ–3ʹ)
*Ndufa7*	CCTGGTGGTCCAGCACATTTAAT	GTTAAATGTGCTGGACCACC
*Ndufs1*	CCGAGATGACCAGAAAAGCACGT	GCGTGCTTTTCTGGTCATCT
*Ndufs3*	GAGGTTCTAATTGCACCTGAGGG	GAGGTTCTAATTGCACCTGA

(C) Primers used for gene expression determination.			
Genes	Primer names	Primer sequences	Sizes of products
*GAPDH*			
(reference gene)	*GAPDH*‐S (5ʹ–3ʹ)	TCGACAAGGCTTCTGCTCACT	146 bp
*Ndufa7*	*GAPDH*‐A (3′–5′)	GGTGCAAGAGGCATTAGATACGA	
	*Ndufa7*‐S (5ʹ–3ʹ)	CTGACAAGGGCACCGCTAA	252 bp
	*Ndufa7*‐A (3′–5′)	TGCCGACTGTGCGAGTTGTA	

**Cloning and phylogenetic analysis** The PCR amplification products were separated using 1.0% agarose gel electrophoresis, and the target amplicons were purified using a TaKaRa MiniBEST Agarose Gel DNA Extraction Kit (Takara, Dalian, China). Purified products were subsequently inserted into the pBlueScript SK vector (ZEYE, Shanghai, China) for sequencing. Amino acid sequences of the coding region (CDS) of *B. tau* for each CI gene were isolated.

The amino acid sequences of the 3 CI genes in the CDS region were subjected to homology analysis with those of other species using the National Center for Biotechnology Information (NCBI) PROTEIN BLAST (Basic Local Alignment Search Tool) to ensure 3 genes before gene knockout. The sequences were aligned using ClustalW, and a maximum likelihood phylogenetic tree was constructed based on the evaluated Dayhoff matrix model using MEGA v7.0 (Kumar *et al.*, 2016) with 1 000 bootstrap replicates.

**Fig. 1:**
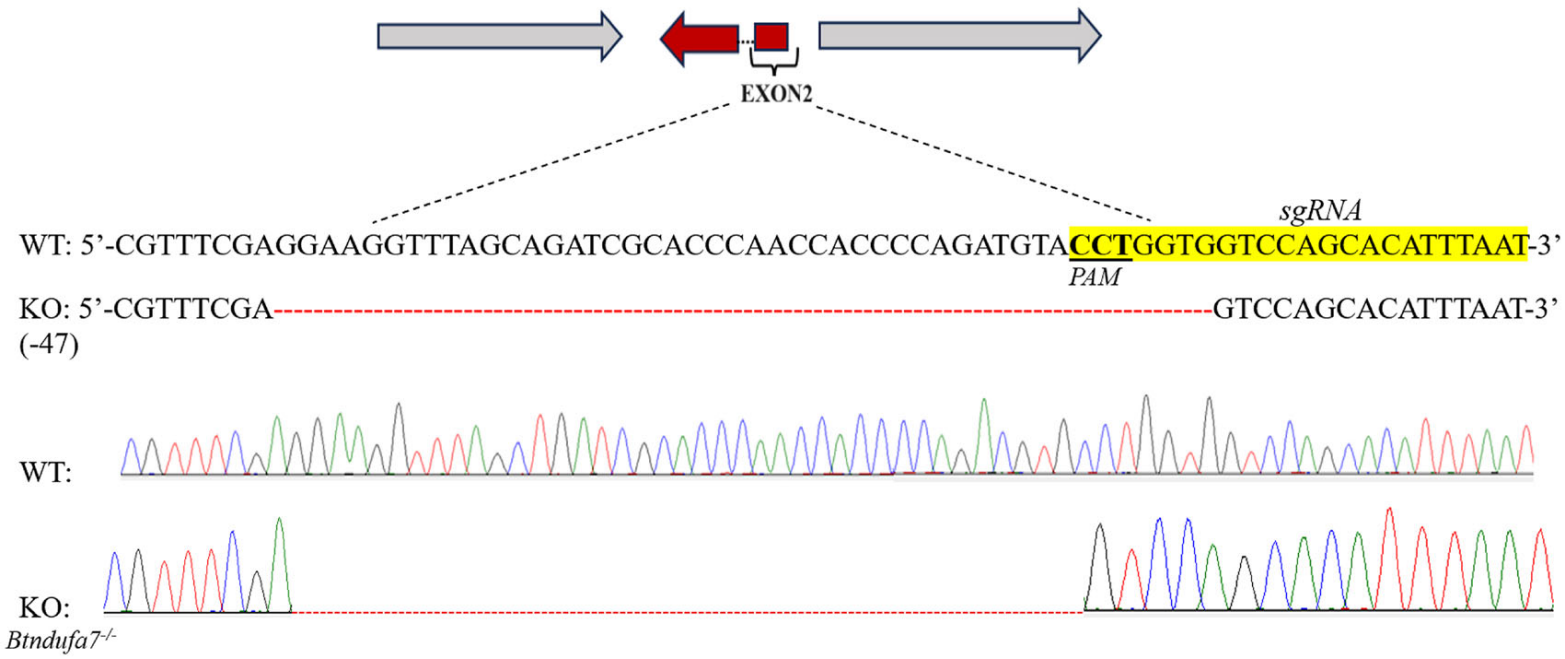
Information about the *Ndufa7* strain of *Bactrocera tau*. Single guide RNA (sgRNA) target region is in exon 2. Yellow highlighting shows the sgRNA target sequence, and the 3 underlined bases are the protospacer adjacent motif (PAM) sites. WT represents the wild‐type sequence and its chromatogram. KO represents the mutational sequence of the F3 homozygotes (*Bndufa7* ^−/−^) and its chromatogram. The red dotted lines represent the deleted bases (−47).

**Fig. 2:**
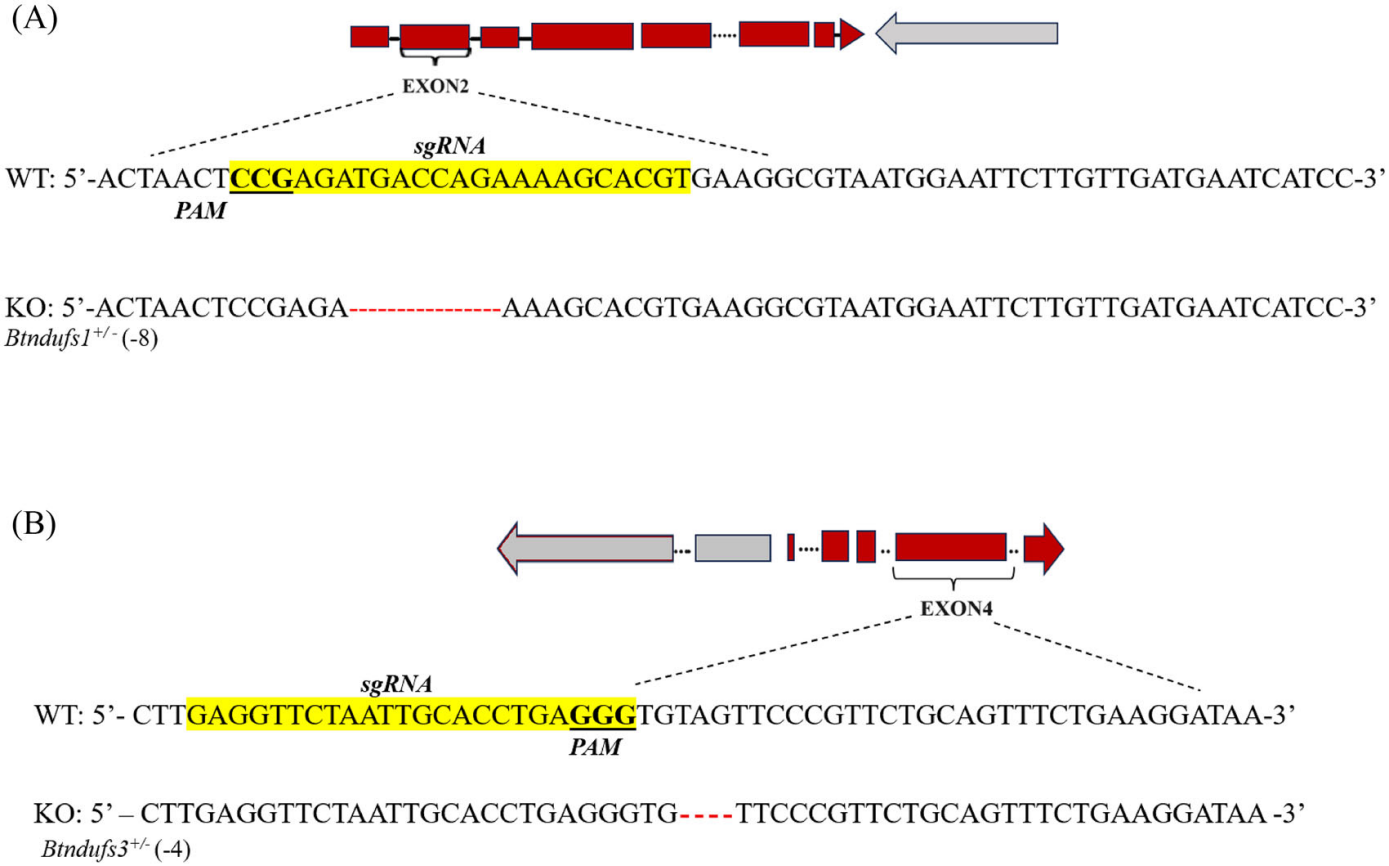
Information about the *Ndufs1* and *Ndufs3* strains of *Bactrocera tau*. (A) Single guide RNA (sgRNA) target region of *Ndufs1* is in exon 2. WT represents the wild‐type sequence, and KO represents the mutational sequence of the F3 heterozygotes (*Bndufs1* ^+/−^) after the *Ndufs1* knockout; (B) sgRNA target region of *Ndufs3* is in exon 4. WT represents the wild‐type sequence, and KO represents the mutational sequence of the F3 heterozygotes (*Bndufs3* ^+/−^) after the *Ndufs3* knockout. The yellow highlights represent the sgRNA target sequences, and the 3 underlined bases are the protospacer adjacent motif (PAM) sites. The red dotted lines represent the deleted bases (−8 for *Bndufs1* ^+/−^, −4 for *Bndufs3* ^+/−^).

**Gene knockout ribonucleoprotein (RNP) constructs** Three Cas9 (clustered regularly interspaced short palindromic repeats [CRISPR]‐associated nuclease 9) – sgRNA (single guide RNA) RNP complexes were constructed separately to knock out each CI gene. Each RNP comprised a Cas9 protein and a sgRNA component. Each sgRNA was designed separately based on the sequences of the coding regions of each gene using the online tool CRISPR RGEN (http://crispor.tefor.net/). The 3 knockout sites are shown in Figs. 1 and 2. The sgRNA sites and protospacer adjacent motif (PAM) of each gene were designed to target exon 2 of *Ndufa7* (marked in Fig. 1) and *Ndufs1* and exon 4 of *Ndufs3* (marked in Fig. 2). The sgRNA targets and sequences are listed in Table 1B. Three sgRNAs were produced with a MEGAshortscrip T7 Transcription Kit (Thermo Fisher Scientific, Vilnius, Lithuania), purified via a GeneArt Precision sgRNA Synthesis Kit (Invitrogen, Vilnius, Lithuania), and stored at −80 °C. The corresponding Cas9 protein (Invitrogen, Vilnius, Lithuania) was mixed with the sgRNAs and incubated at 37 °C for 30 min to form 3 independent Cas9‐sgRNA RNP (300 ng/*µ*L sgRNA and 500 ng/*µ*L Cas9 protein).

**Three strains of *B. tau* after gene knockout** Approximately 1 500 eggs collected from wild‐type females of *B. tau* after mating were used for Cas9‐sgRNA RNP injection. The eggs were cleaned with ultrapure water and then washed in a 1% sodium hypochlorite solution to remove the chorion. The eggs were placed in rows on microscope slides for microinjection. The eggs were covered with Halocarbon oil 700 (Sigma, MO, USA) before injection. Each of the 3 Cas9‐sgRNA RNP was injected into approximately 500 eggs. A microinjection system (Drummond, AL, USA) was used to inject RNP into the posterior pole of each egg within 30 min of egg laying. The injected eggs were then placed 3 isolated larval incubators (1 each RNP) for hatching. The larvae in each incubator were then transferred to 3 separate cages for adult emergence. Three gene strains of *B. tau* were established in 3 isolated cages and named *NA7*‐KO for the *Ndufa7* knockout strain, *NS1*‐HT and *NS3*‐HT for *B. tau* strains after *Ndufs1* and *Ndufs3* knockout, respectively. The wild‐type strain of *B. tau* was named BAT‐WT.

Adults emerging from the injected eggs were regarded as zero generation (F0) for each strain. The F0 generation flies were crossed with wild‐type flies (WT). DNA was extracted from the whole bodies of F0 flies using a TiANamp Genomic DNA Kit (TIANGEN, Beijing, China), after which PCR amplification and sequencing were performed. PCR amplification was performed under the conditions described above (see “*Knockout gene selection and PCR*”). F0 fly genotypes were identified, and flies harboring mutations and WT alleles were retained. After the F0 flies of each strain laid eggs that hatched into the F1 generation, the F1 generation flies of each strain were crossed with BAT‐WT. Similarly, F1 flies laid eggs, and their genotypes were identified using sequencing. F1 generation flies with 2 types of alleles were maintained and their F2 generation offspring were crossed with one another. Individuals of the F3 generations were obtained, and F3 individuals, including dead and living individuals, were sequenced. As many homozygous flies as possible were obtained for each gene strain after identifying the F3 flies.

### Fitness estimation

**Host plants determination** We screened host fruits to examine whether the host fitness of *B. tau* reared on various host fruits changed before and after gene knockout (3 CI genes). Based on previous reports (Huang *et al.*, 2005; Lin *et al.*, 2005; Hasyim *et al.*, 2008; Zhang & Chen, 2018; Shi *et al.*, 2020a; Shi *et al.*, 2022b) and odor attraction test of some fruits (see Fig. S1), 2 native cucurbit hosts (summer squash, *Cucurbita pepo* var. fastigata L.; cucumber, *Cucumis sativus* L.), and 2 novel hosts (banana, *Musa paradisiaca* Colla; guava, *Psidium guajava*) were tested first. Pitaya (*Hylocereus undulatus* Britt) and apple (*Malus pumila* Million, a temperate fruit) were hypothesized to be potential hosts of *B. tau* based on our odor attraction test (Fig. S1). In the present study, we attempted to estimate the possibility of *B. tau* expanding to 2 hosts. Notably, although apple odors attracted *B. tau*, we found that *B. tau* laid very few eggs on apples and *B. tau* did not rear on apples continuously after actual rearing of *B. tau* on this host. Therefore, we did not use apples in subsequent tests. Therefore, summer squash, cucumber, banana, guava, and pitaya fruits were used to continuously evaluate the fitness of *B. tau*.

**Fitness estimation before gene knockout** We first performed a fitness evaluation of BAT‐WT in the 5 hosts previously mentioned. In total, 150 BAT‐WT adults reared on pumpkin fruits were evenly transferred to 5 cages with different host fruits. Each cage contained 30 BAT‐WT adults, including 15 females and 15 males. Slices and whole fruits were provided to *B. tau* for feeding and laying eggs, respectively, in each cage. The *B. tau* adults were reared continuously for at least 3 generations on different hosts.

Three indices, including pupal number (PN), single‐pupal weight (SG), and emergence rate (ER), were used to estimate the fitness of *Z. cucurbitae*, *Bactrocera zonata* (Saunders), and *B. tau* on different hosts (Hafsi *et al.*, 2016; Shi *et al.*, 2017c). The mean values of each index from 3 generations (F3–F5) of each host were determined.

**Fitness estimation after gene knockout** We obtained a stable homogenous F3 generation only in *NA7*‐KO after gene knockout using the CRISPR‐Cas9 system. Homogenous lethality occurred in both the *Ndufs1* and *Ndufs3* strains. Therefore, we only estimated the fitness of the *NA7*‐KO in the 5 hosts. Adults from the F3 generation of *NA7*‐KO were transferred to 5 different expansion phases of host plants for at least 3 generations of rearing. Next, the mean values of the indices (PN, SG, and ER) from 3 continuous generations (F3–F5) were calculated to estimate host fitness for *NA7*‐KO on 5 host plants.

### Relative expression levels of the *Ndufa7* gene

Given the homogenous F3 generation of *Ndufa7 B. tau* strains, the relative gene expression levels of *Ndufa7* were tested before and after the gene knockout. We first examined whether the relative expression of *Ndufa7* in BAT‐WT changed when *B. tau* flies were fed native (summer squash and cucumber), novel (banana and guava) or potential host fruits (pitaya). Next, we examined whether the relative expression of the *Ndufa7* gene of *NA7*‐KO changed when *B. tau* fed on the various hosts. This examination was conducted using quantitative real‐time PCR (qPCR) according to the manufacturer's instruction by using the StepOnePlus Real‐Time PCR System (Thermo Fisher Scientific, MA, USA). Gene‐specific primers are listed in Table 1C. *GAPDH* was used as an internal reference. The 3rd instar larvae of BAT‐WT and *NA7*‐KO reared on various hosts were collected for 3 continuous generations (F3–F5), and the relative gene expression levels of each generation were estimated. The mean values of the relative gene expression levels of BAT‐WT and *NA7*‐KO from continuous generations (F3–F5) were also estimated.

### Statistical analysis

Data are presented as means ± standard error (SEs). The statistical significance of the differences was determined using SPSS v23.0 (SPSS, IL, USA). Differences between fitness indices and gene expression levels pre‐ and post‐gene knockout were compared using unpaired *t*‐tests. Statistical significance was set at *P < 0.05; **P < 0.01; ***P < 0.001.

## Results

### Knockout of 3 CI genes

**Fig. 3:**
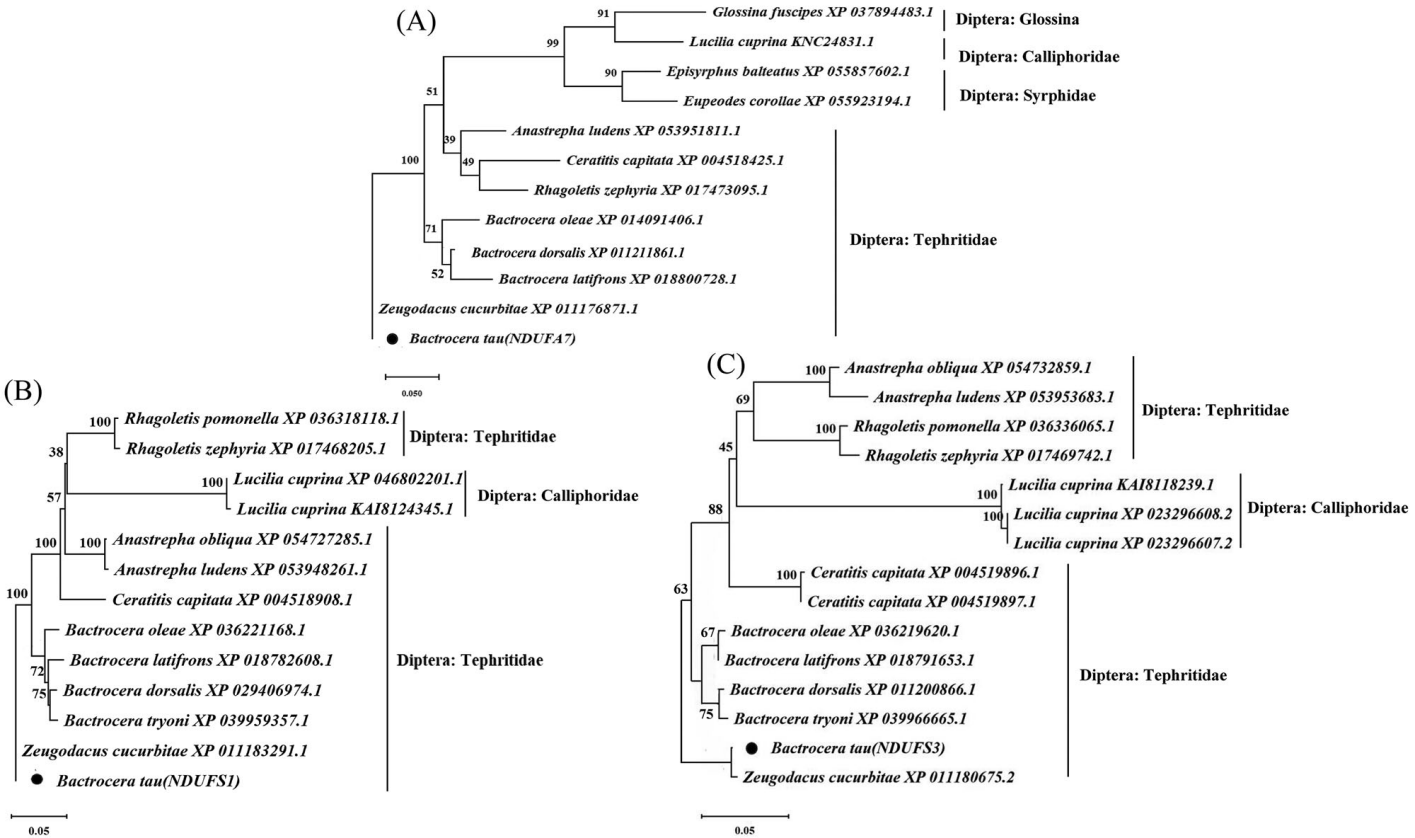
Phylogenetic trees constructed based on the results of homologous comparisons of the 3 complex I (CI) genes from other homologous species of *Bactrocera tau*. (A) Phylogenetic tree of *Ndufa7* amino acid sequences of *B. tau* and its homologous species; (B) phylogenetic tree of *Ndufs1* amino acid sequences of *B. tau* and its homologous species; (C) phylogenetic tree of *Ndufs3* amino acid sequences of *B. tau* and its homologous species.

**Homology comparison** The amino acid sequences of *B. tau* in the coding region of each target gene were obtained, that is, 98, 736 and 264 amino acids in *Ndufa7*, *Ndufs1*, and *Ndufs3*, respectively (Fig. S2). The amino acid sequences of each gene were phylogenetically analyzed and compared with those of other homologous species to ensure the 3 target genes before gene knockout. The amino acid variations in the coding region of each gene of *B. tau* and its top 4 BLAST hits are shown in Fig. S2. The *Ndufa7* amino acid sequence of BAT‐WT was highly homologous to those of other tephritids, such as *Z. cucurbitae*, *Bactrocera dorsalis*, and *Anastrepha ludens* (Loew) (see Figs. 3A and S2). The amino acid sequences of *Ndufs1* and *Ndufs3* in BAT‐WT were highly homologous to those of other tephritid species, including *Bactrocera oleae* (Rossi), *Z. cucurbitae*, *B. dorsalis*, and *Bactrocera tryoni* (Froggatt) (Figs. 3B,C, and S2). These results suggested that the 3 target genes belong to the mitochondrial CI of tephritids (Diptera).

**ER and *B. tau* strain identification** After gene knockout, pupae of the F3 generation of each strain were obtained through several rounds of crossing using the F0–F2 knockouts. The emergence rates of the F3 pupae were 54%, 23%, and 30% for *NA7*‐KO, *NS1*‐HT, and *NS3*‐HT, respectively.

The enclosed F3 adults of the *NA7*‐KO were identified as homozygous (*Btndufa*7^−/−^) based on sequencing chromatograms (Figs. 1 and S3). It showed that the *NA7*‐KO F0 to F2 individuals were heterozygous, but the F3 individuals were homozygous (unimodal chromatograms). However, the individuals of the *NS1*‐HT and *NS3*‐HT from the F0 to F3 generations were identified as heterozygous (*Btndufs1* ^+/−^ and *Btndufs3* ^+/−^, multimodal chromatograms) without homozygous individuals, as shown in Fig. S3. Some dead F3 pupae or adults of *NS1*‐HT and *NS3*‐HT were randomly selected for sequencing. Most of the sequences from dead *B. tau* were homozygous according to unimodal chromatograms (74% of F3 dead flies of *NS1*‐HT and 82% of F3 dead flies of *NS3*‐HT). Therefore, homozygous lethality occurred in *NS1*‐HT and *NS3*‐HT.

**Deleted nucleobases and phenotype variation** The deleted parts of the 3 strains were evaluated after gene knockout (Figs. 1 and 2). In total, 47, 8, and 4 bp nucleobases of *Ndufa7*, *Ndufs1*, and *Ndufs3*, respectively, were deleted.

**Fig. 4:**
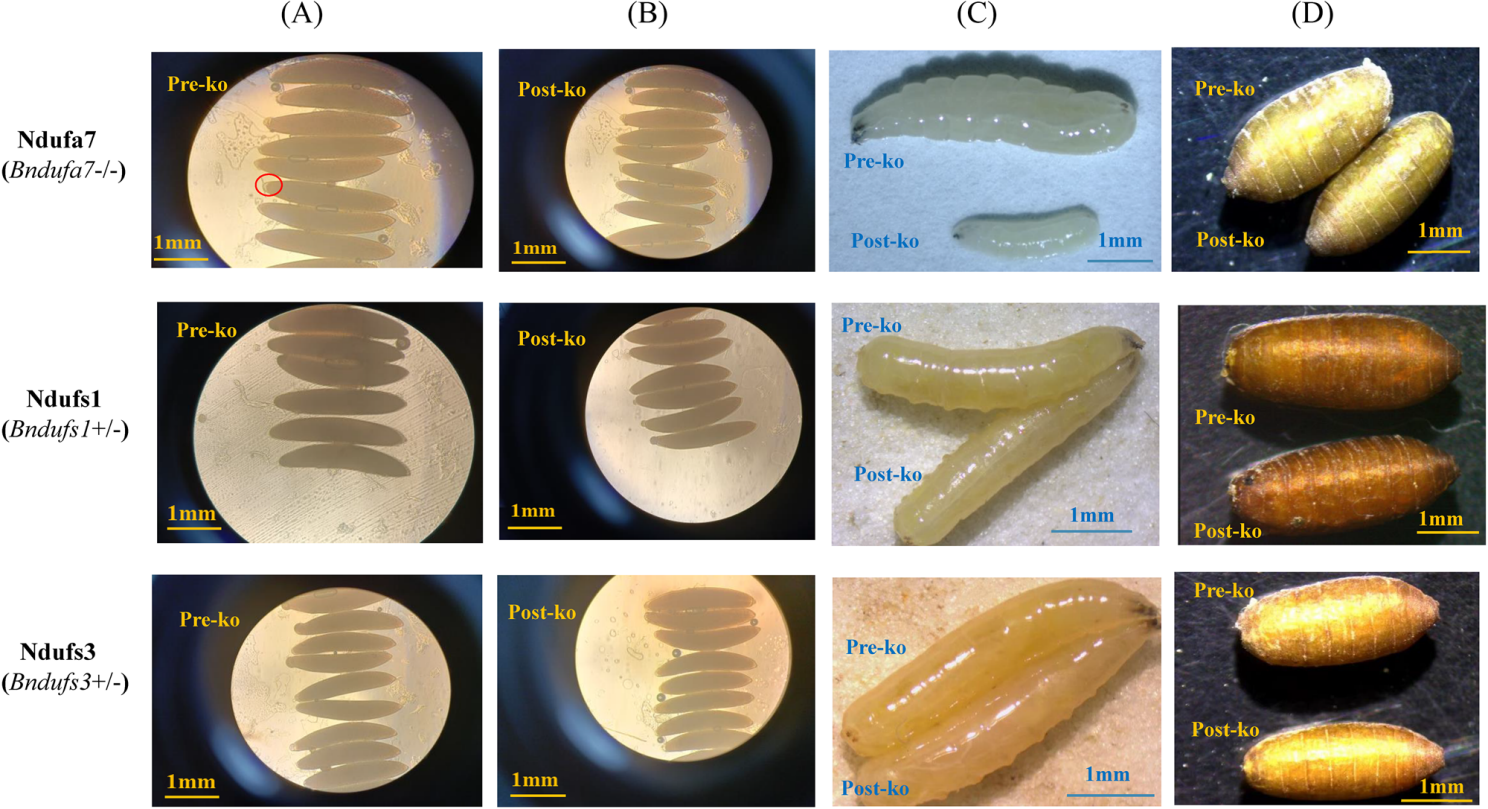
Comparison of the *Bactrocera tau* phenotypes pre‐ and post‐knockout. (A) Eggs of *B. tau* pre‐knockout (red circle indicating young layer); (B) eggs of *B. tau* post‐knockout; (C) comparison of larvae pre‐ and post‐knockout. The upper panel shows the larvae of the wild‐type, and the lower panel shows the larvae of the strains after post‐knockout. (D) Comparison of pupae pre‐ and post‐knockout. The upper panel shows pupae of the wild‐type, and the lower panel shows pupae of the strains post‐knockout. A scale bar is provided in each image. *Ndufa7* (*Bndufa7* ^−/−^), *Ndufs1* (*Bndufs1* ^+/−^), and *Ndufs3* (*Bndufs3* ^+/−^) are 3 complex I (CI) genes (gene type for *B. tau* of F3 generation post‐knockout).

Eggs with young layers (Fig. 4A) were selected to be injected with knockout RNP because they are more active. There were no apparent differences in the phenotypes of eggs before and after the knockout among the 3 strains (Fig. 4A, B). However, differences in phenotypes were observed between larvae and pupae pre‐ and post‐knockout (Figs. 4C,D). In particular, the post‐knockout larvae and pupae from the *NA7*‐KO F3 generation were significantly smaller than those from the pre‐knockout generations. However, no noticeable phenotypic differences were observed in larvae or pupae post‐knockout for the other 2 gene strains (*NS1*‐HT and *NS3*‐HT).

### Fitness variation in *Ndufa7* pre‐ and post‐knockout

Fitness was estimated using 3 fitness indices (SG, PN, and ER) for *B. tau* reared on native, novel, and potential hosts pre‐ and post‐knockout of the *Ndufa7* gene.

**Fig. 5:**
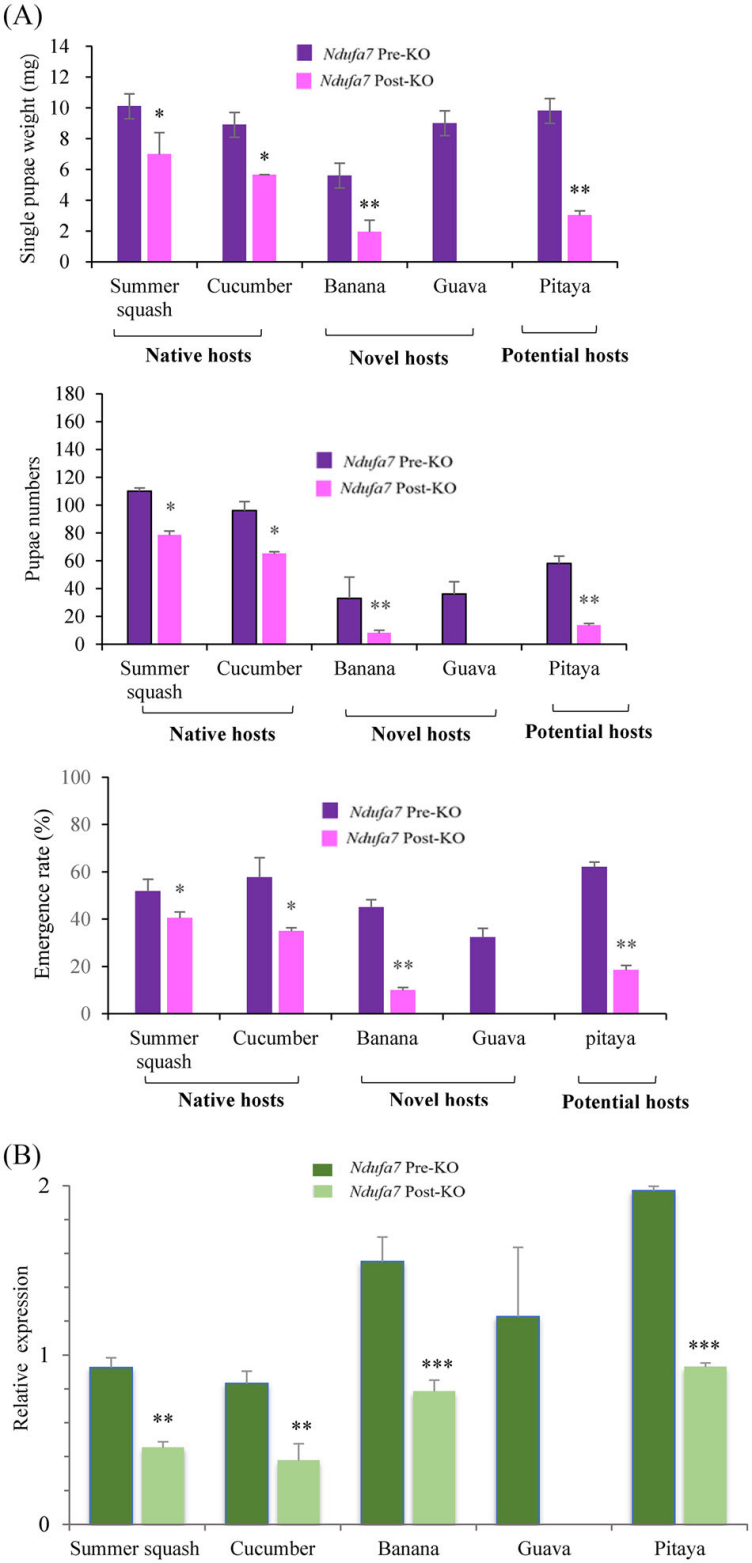
Fitness variation and relative gene expression levels of *Ndufa7*. (A) Fitness of *Bactrocera tau* on various hosts. *Ndufa7* pre‐knockout (pre‐KO) (purple columns): mean values of each index (single‐pupal weight [SG], pupal number [PN] and emergence rate [ER]) from continuous 3 generations (from F3 to F5) of *B. tau* before *Ndufa7* knockout. *Ndufa7* Post‐KO (pink columns): mean values of each index from continuous 3 generations (from F3 to F5) *NA7*‐KO of *B. tau*. Significant differences between mean values of *Ndufa7* pre‐KO and *Ndufa7* post‐KO were estimated by *t*‐test and indicated by asterisks (^*^ *P *< 0.05; ^**^ *P *< 0.01). Note: *NA7*‐KO cannot rear on guava at all (no data). (B) Mean values of relative gene expression levels of *Ndufa7*. Given only homogenous F3 generation of *Ndufa7 B. tau* strains, relative expression levels of the *Ndufa7* gene were tested before and after *Ndufa7* knockout. *Ndufa7* pre‐KO (dark green columns): means of *Ndufa7* expression levels of *B. tau* larvae from continuous 3 generations (F3–F5) that were reared on 5 various hosts before *Ndufa7* knockout. *Ndufa7* post‐KO (light green columns): means of *Ndufa7* expression levels of *B. tau* larvae from continuous 3 generations (F3–F5) that were reared on 4 hosts after *Ndufa7* knockout. Differences between mean values of *Ndufa7* pre‐KO and *Ndufa7* post‐KO were estimated using a *t*‐test. The significance is indicated by asterisks (**P < 0.01; ***P < 0.001). Note: *NA7*‐KO did not survive on guava (no data).

First, the 3 fitness indices showed that both BAT‐WT and *NA7*‐KO flies could continuously rear well on 2 native cucurbit hosts when transferred from the native pumpkin host (Fig. 5A). However, the 3 fitness indices of *NA7*‐KO reared on the 2 native hosts decreased slightly compared with that of *NA7*‐WT (*P* < 0.05, Fig. 5A), suggesting that *Ndufa7* knockout impaired the adaptability of *B. tau* to spread to the 2 native hosts. Second, the 3 fitness indices showed that BAT‐WT flies reared well on novel hosts banana and guava for continuous generations (Fig. 5A). *NA7*‐KO flies could still continuously rear on banana. However, there were significant decreases in the fitness indices of *NA7*‐KO reared on banana compared with those of BAT‐WT (*P* < 0.01, Fig. 5A). Furthermore, *NA7*‐KO could not rear guava at all, thus lacking fitness data (Fig. 5A). These results suggested that *Ndufa7* knockout largely impaired the adaptability of *B. tau* to the 2 novel hosts after expansion from the pumpkin host. Finally, BAT‐WT and *NA7*‐KO flies could continuously rear well on a potential host, pitaya. However, there were significant decreases in the fitness indices of *NA7*‐KO compared with those of BAT‐WT (*P* < 0.01, Fig. 5A), indicating that *Ndufa7* knockout had a large effect on the adaptability of *B. tau* to a potential host, that is, pitaya, after host expansion from pumpkin hosts.

Therefore, the *Ndufa7* gene was involved in the survival of *B. tau* on native hosts and in the expansion of *B. tau* to novel and potential hosts.

### Relative expression levels of *Ndufa7* gene

We also examined the relative gene expression levels of *Ndufa7* following *B. tau* rearing on native, novel, and potential hosts pre‐knockout of the *Ndufa7* gene to further understand the role of *Ndufa7* in host expansion of *B. tau*. However, *NA7*‐KO did not survive on guava; therefore, we did not test *Ndufa7* expression in *NA7*‐KO on guava (no data) but only on the 4 remaining hosts (summer squash, cucumber, banana, and pitaya) after *Ndufa7* knockout.

First, the *Ndufa7* gene was expressed in BAT‐WT and *NA7*‐KO flies reared on 2 native cucurbit hosts (summer squash and cucumber). However, there were decreases in NA7‐KO when reared on summer squash and cucumber compared to that in BAT‐WT (*P* < 0.01, Fig. 5B), suggesting that *Ndufa7* was involved in the adaptation of *B. tau* to 2 native hosts, expanding from the native pumpkin host. Second, *Ndufa7* was highly expressed in BAT‐WT flies reared on novel banana and guava hosts. However, there were significant differences in *Ndufa7* expression levels between NA7‐KO and BAT‐WT flies reared on banana (*P* < 0.001; Fig. 5B). These results suggested that the *Ndufa7* gene affected the adaptation of *B. tau* to novel hosts, expanding from the native pumpkin host. Lastly, *Ndufa7* can be expressed in both BAT‐WT and *NA7*‐KO flies reared on a potential host, pitaya, when transferred from native pumpkin fruits. However, there were sharp decreases in relative *Ndufa7* expression levels in NA7‐KO flies compared with that in BAT‐WT flies (*P* < 0.001, Fig. 5B), indicating that the *Ndufa7* gene is involved in the host expansion of *B. tau* to this potential host. In summary, different *Ndufa7* expression levels in *B. tau* pre‐ and post‐gene knockouts indicated that *Ndufa7* is involved in the survival of *B. tau* on native hosts and in the expansion of *B. tau* to novel and potential hosts.

## Discussion

We revealed the respective roles of *Ndufa7*, *Ndufs1*, and *Ndufs3* in host expansion of the invasive pest *B. tau* for the first time. *Ndufa7* knockout interfered with *B. tau* survival on native hosts and disrupted *B. tau* expansion to novel and potential hosts. However, knockout of *Ndufs1* and *Ndufs3* caused homogenous lethality in *B. tau*.

### Role of 3 CI genes in host expansion of *B. tau*

In this study, stable homogenous *NA7*‐KO (*Btndufa7* ^−/−^) *B. tau* was established after *Ndufa7* knockout. However, compared with that of BAT‐WT, the size of the larvae and pupae of *NA7*‐KO shrank, suggesting that *Ndufa7* had a small effect on the development of *B. tau*. Furthermore, in contrast to BAT‐WT, *NA7*‐KO did not survive on the novel host guava. *NA7*‐KO also performed poorly on the novel host banana and potential host pitaya, as shown by sharp decrease in fitness and *Ndufa7* gene expression levels. The ability of *NA7*‐KO to adapt to 2 native hosts, summer squash and cucumber, was slightly reduced in terms of fitness and relative *Ndufa7* expression levels. Therefore, *Ndufa7* is involved in the early development and survival on native hosts and later expansion to novel hosts and potential hosts in *B. tau*.

Silencing *Ndufa7* caused variations in the levels of both host fitness and *Ndufa7* expression in *B. tau* expanding to native, novel and potential hosts. Based on this evidence, we speculated that the effects of different levels on the host expansion of *B. tau* caused by *Ndufa7* were associated with the host chemical environment. When *B. tau* was expanded to 2 cucurbit hosts, similar host environments stimulated slight responses of *Ndufa7*, which resulted in a gentle variation in fitness and gene expression patterns after *Ndufa7* knockout. In contrast, *B. tau* encounters very different chemical environments when it expands to novel and potential hosts belonging to different plant families and genera. Guava is a plant of the pomegranate genus, banana is a plant of the *Musa* genus (Shi *et al.*, 2022b), and pitaya is a plant of the *Selenicereus* genus (Shan *et al.*, 2023). *B. tau* must spend more energy to detoxify and digest various secondary metabolites and stimulate a strong *Ndufa7* response to adapt to new chemical challenges, resulting in large variations in fitness and *Ndufa7* expression levels. Therefore, the chemical environment of the host is a key factor driving *Ndufa7*, which plays a role in the host expansion of *B. tau*. The influence of the host chemical environment in *B. tau* before gene knockout has been shown with a potential host, that is, apples. *B. tau* could lay a few eggs on apples, but they could not become adults. However, *B. tau* could successfully expand on another potential host, pitaya, although it has a similar odor attraction with that of apples (Fig. S1). *B. tau* cannot adapt to the chemical environment of apples (*Rosa* genus) (Zhang & Chen, 2018), whereas flies can adapt to the chemical environment of pitaya.

Only limited information on the role of *Ndufa7* is available (Xiao *et al.*, 2022). Several studies have shown that *Ndufa7* is associated with cardiac hypertrophy (Shi *et al.*, 2020) and rheumatoid arthritis (Mitsunaga *et al.*, 2015). *Ndufa7* plays a fundamental role in the pathogenesis of these diseases by regulating oxidative stress, which is characterized by the accumulation of ROS (Shi *et al.*, 2020). Overaccumulation of ROS affects the production of adenosine triphosphate (ATP) and the energy supply (Lin *et al.*, 2021), which causes disease. For example, the depletion of *Ndufa7* promotes increasing ROS production and calcineurin signaling activation, contributing to cardiac hypertrophy (Shi *et al.*, 2020). Similarly, excessive ROS levels drive rheumatoid arthritis (Mitsunaga *et al.*, 2015) associated with *Ndufa7*. Thus, we speculated that *Ndufa7*, which affected native host adaptation and novel and potential host expansion in this study, is probably associated with an oxidase‐stressing mechanism. Due to chemical differences between native, novel and potential hosts (chemical stressors), *Ndufa7* may regulate ROS production and ATP formation. Thus, the dysfunction of *Ndufa7* probably causes variations of ROS and ATP, leading to different “behavioral symptoms” of *B. tau* in response to 5 hosts. Therefore, future studies should explore the ROS metabolism of host expansion in *B. tau*.

Previous studies have shown that *Ndufs1* and *Ndufs3* genes are associated with several diseases such as cancer (Kurelac *et al.*, 2019; Ren *et al.*, 2023), Leigh syndrome (Peralta *et al.*, 2020), mitochondrial disease (Granat *et al.*, 2024), and Alzheimer's disease (Lin *et al.*, 2021). The dysfunction of these 2 genes causes apoptosis, respiratory chain defects, early mortality, and a decline in ATP production (Elkholi *et al.*, 2019; Kurelac *et al.*, 2019; Peralta *et al.*, 2020; Lin *et al.*, 2021; Ren *et al.*, 2023; Granat *et al.*, 2024).

Stable homogenous strains were not established after silencing *Ndufs1* and *Ndufs3* in the present study. However, heterozygous strains (*Btndufs1* ^+/−^ and *Btndufs3* ^+/−^) of *B. tau* were obtained after gene knockout, exhibiting homozygous lethality for the 2 gene strains. Homozygous lethality mainly occurred during the embryogenesis stage, which is the first report of tephritid pests of the *Ndufs1* and *Ndufs3* genes in this study. Therefore, *Ndufs1* and *Ndufs3* play a role in the embryonic development of *B. tau*. Our results provide new evidence supporting the role of these 2 core genes in development.

*Ndufs1* and *Ndufs3* are core structure units of CI, and they represent the N and Q functional modules of CI, respectively (N module functions in oxidizing nicotinamide adenine dinucleotide hydrogen and Q module functions in reducing ubiquinone). They play important roles in the assembly and structural stability of the CI (Stroud *et al.*, 2016; Su *et al.*, 2016). Thus, the knockout of *Ndufs1* or *Ndufs3* resulted in *B. tau* homogenous lethality in our study. However, *Ndufa7* is an accessory unit of the CI (Hirst *et al.*, 2003; Xiao *et al.*, 2022). Previous studies have shown that *Ndufa7* knockout did not affect the assembly and structure of CI, but had a mild effect on CI activity (Stroud *et al.*, 2016). *Ndufa7* knockout also did not alter cell viability (Xiao *et al.*, 2022). This suggests that *Ndufa7* may have a regulatory role in CI activities, such as ATP production and ROS variation. Indeed, our results suggest that *Ndufa7* can regulate early development and survival on hosts and the later expansion to novel and potential hosts of *B. tau*.

### Potential of 3 CI genes in future RNAi control

For tephritid flies, environmentally friendly management methods such as RNAi‐based (Shelly *et al.*, 2014) control methods have been widely suggested (Zhu & Palli, 2020). Some genes related to sex determination (*Astra‐2*, *tra‐2* gene) (Li & Handler, 2019), pupal color (*wp* gene) (Ward *et al.*, 2021), and temperature sensitivity (*shibire* gene) (Choo *et al.*, 2020) have been exploited and applied in RNAi‐based control methods for *Ceratitis capitate* (Widedmann), *B. dorsalis*, (Sim *et al.*, 2019) and *B. tryoni* (Choo *et al.*, 2020). However, only a few genes involved in host expansion have been targeted using RNAi‐based control. We identified the functions of these 3 CI genes in *B*. tau host expansion in the present study. Given the regulatory role of Ndufa7 in native host selection and novel and potential host expansion, it should be considered a potential target gene for RNAi control in tephritids. The other two genes were not suitable targets for RNAi‐based controls because of their lethality upon homozygous knockout. In fact, mitochondrial CI genes have wide potential for pest control, and several studies have reported lepidopteran pest control. For example, the use of transgenic cotton tissues expressing *Ndufv2* double‐stranded RNA (dsRNA) to treat the cotton bollworm (*Helicoverpa armigera*) larvae led to targeted gene silencing, resulting in high pest mortality (Wu *et al.*, 2016).

In summary, our study provides a new case for verifying the regulatory function of the *Ndufa7* gene for survival and reports a new role of the *Ndufa7* gene in novel and potential host expansion in tephritids for the first time. In contrast, *Ndufs1* and *Ndufs3* played key roles in the early development of *B. tau*. Therefore, only *Ndufa7* is a target gene for RNAi‐based control techniques.

## Disclosure

The authors declare no conflict of interest.

## Supporting information




**Fig. S1** Odor attraction tests of *Bactrocera tau* toward various host fruits via a Y‐tube olfactometer.

**Fig. S2** *Bactrocera tau* amino acid sequences variations in the coding sequence (CDS) region of 3 complex I (CI) genes.

**Fig. S3** Chromatograms for individual *Bactrocera tau* strains randomly selected from the F0 to F3 generations for each complex I (CI) gene strain after knockout.
