# Supplementary material for: Role of mitochondrial complex I genes in host plant expansion of Bactrocera tau (Tephritidae: Diptera) by CRISPR/Cas9 system
Source: Insect Sci. 2025 Jan 19;33(1):147–58. doi: 10.1111/1744-7917.13495 (PMC12905475; doi:10.1111/1744-7917.13495)
Supplement: Supplementary file 1 — Fig. S1 Odor attraction tests of Bactrocera tau toward various host fruits via a Y‐tube olfactometer. [file INS-33-147-s003.pdf]

Fig. S1 Odor attraction tests of *B. tau* toward various host fruits via a Y-tube olfactometer. A. Simplified device diagram of Y-tube olfactometer and detailed processes of test. **B.** Mean numbers of odor attraction test of *B. tau* on various hosts. Differences between two means were estimated by t-test. Significance is indicated by different lowercase letters ($P < 0.05$).

A.

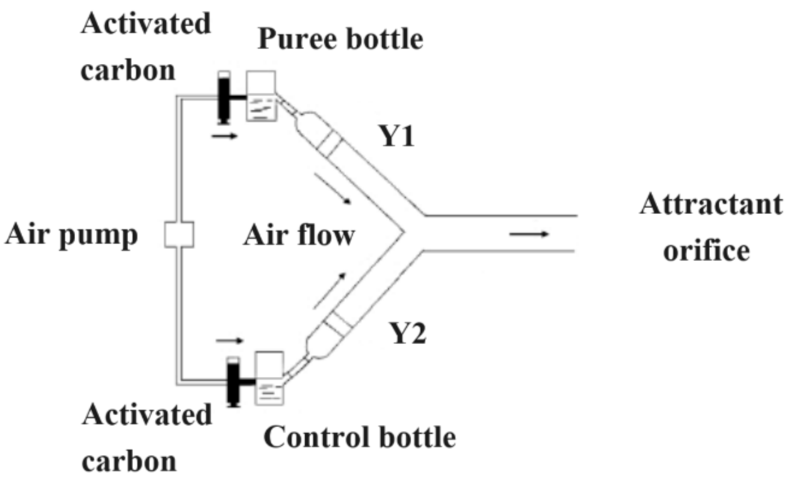

Note: The Y olfactometer consisted of a Y tube, an air pump, an activated carbon device, a humidifier, two sample bottles, two gas flow meters and some connection tubes. Before tests were conducted, the Y tube was washed with ethanol, and the Y olfactometer was assembled. For each test, the puree of one host fruit (approximately 25 mg) was put at the bottom of one sample bottle connecting to one arm of Y tube (Y1), and another sample bottle was as a blank control connecting to another arm of Y tube (Y2). Then, the air pump was opened for approximately 10-15 seconds to ensure air flow (0.5 L/min) through the bottles, and 5 *B. tau* adults were placed at the entrance of the Y tube. We observed the behavior of the adults for 5~10 minutes. If the adults entered the 1/3 arm of the Y1, we regarded the *B. tau* strain as selecting the host. However, if the adult entered the Y2, we considered the *B. tau* strain not to select the host. The odor preference test for each host plant was repeated 5 times, after which the average number and total numbers of *B. tau* in the Y tube were counted. Flies with no any choice did not be counted. When making fruit puree, we chose physiologically mature fruits and puree contain peel and pulp.

B.

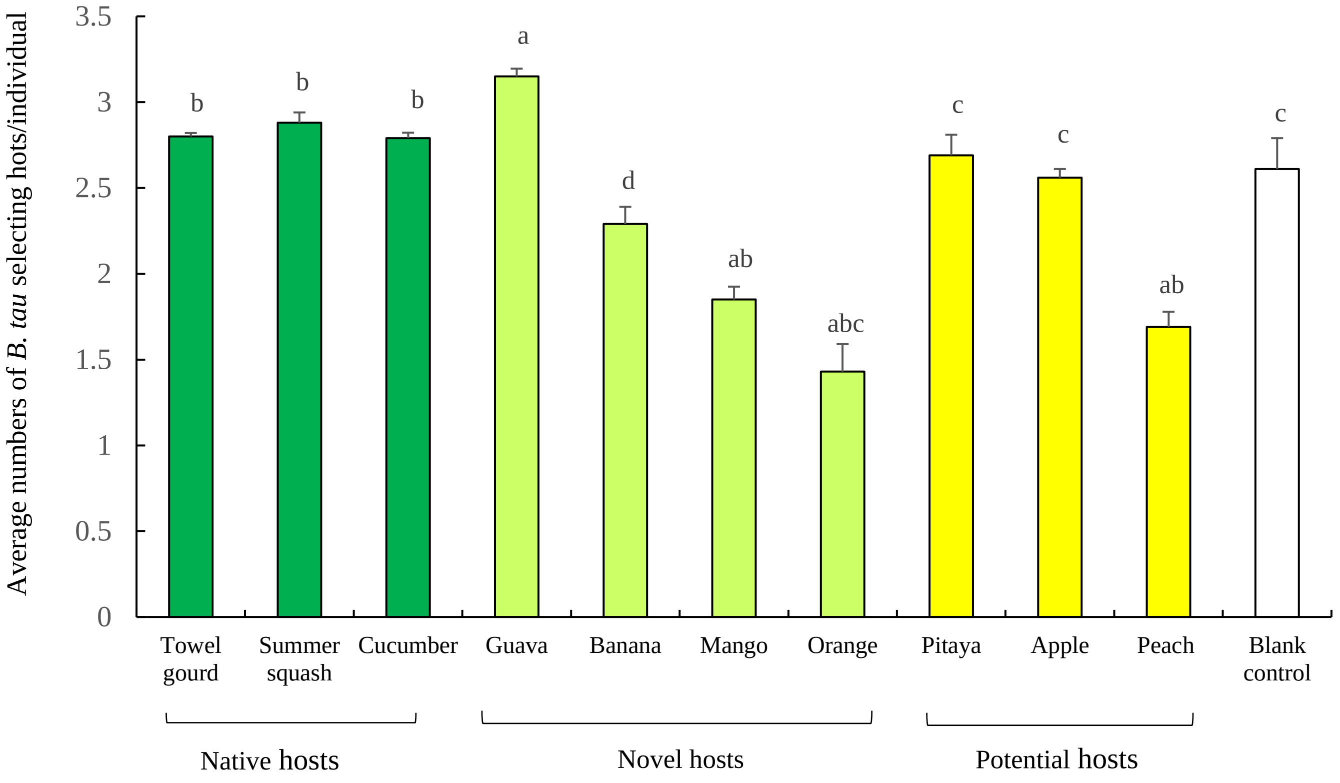

Note: To help determining novel and potential hosts for the fitness evaluation experiments, we first carried out preliminary odor attraction tests of *B. tau* from 10 host plants via a Y olfactometer. According to the levels of *B. tau* preference (average numbers were shown in vertical axis), we selected two novel hosts (banana, guava) and two potential hosts (pitaya, apple).
