# Supplementary material for: Role of mitochondrial complex I genes in host plant expansion of Bactrocera tau (Tephritidae: Diptera) by CRISPR/Cas9 system
Source: Insect Sci. 2025 Jan 19;33(1):147–58. doi: 10.1111/1744-7917.13495 (PMC12905475; doi:10.1111/1744-7917.13495)

Fig. S2 *Bactrocera tau* amino acid sequences variations in CDS region of three CI genes. The top four blast hits of *B. tau* and its homologous species (GenBank numbers of homologous species have shown in Fig.3) in each gene were shown.

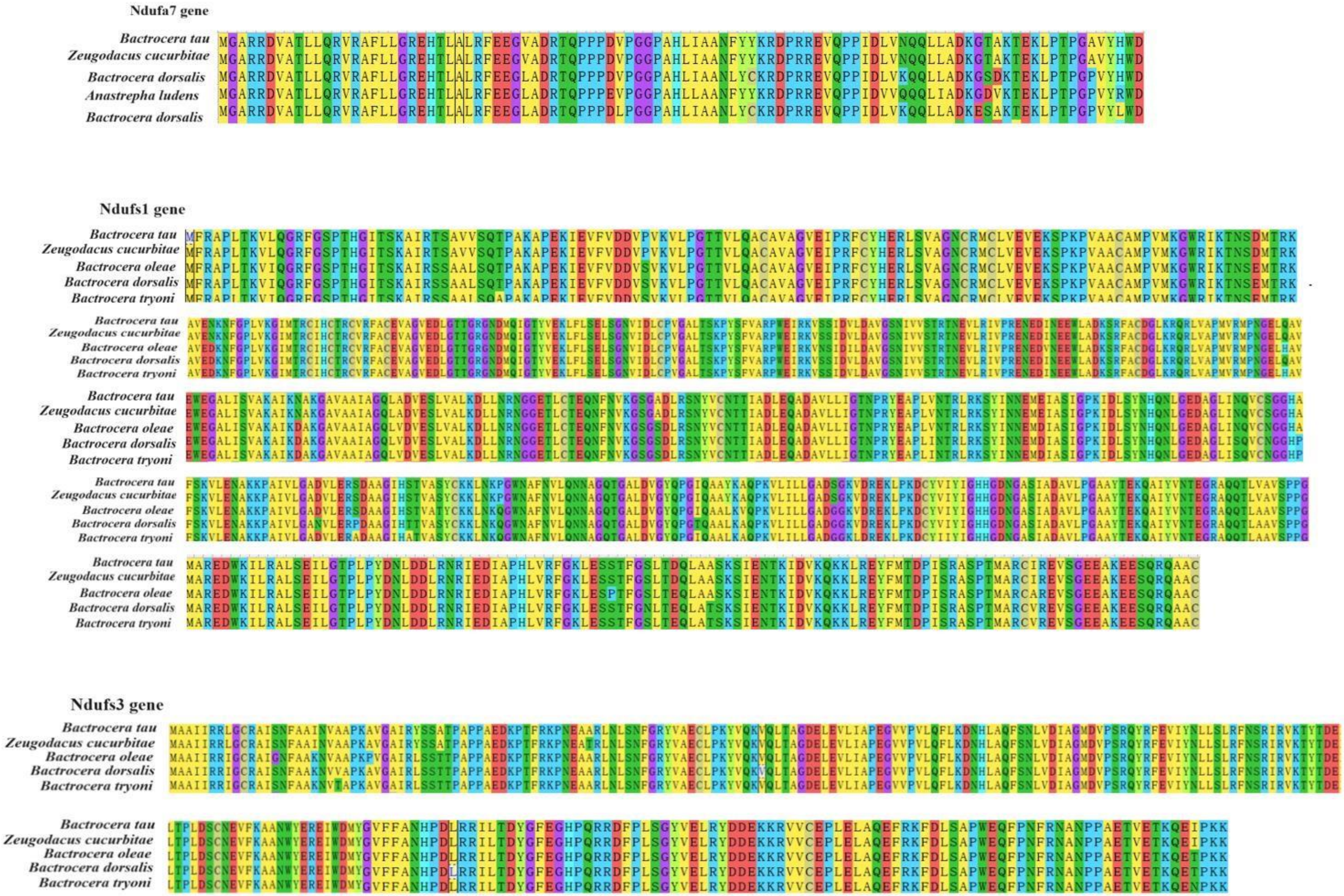

Supplement: Supplementary file 2 — Fig. S2 Bactrocera tau amino acid sequences variations in the coding sequence (CDS) region of 3 complex I (CI) genes. [file INS-33-147-s002.pdf]
