# Supplementary material for: Role of mitochondrial complex I genes in host plant expansion of Bactrocera tau (Tephritidae: Diptera) by CRISPR/Cas9 system
Source: Insect Sci. 2025 Jan 19;33(1):147–58. doi: 10.1111/1744-7917.13495 (PMC12905475; doi:10.1111/1744-7917.13495)
Supplement: Supplementary file 3 — Fig. S3 Chromatograms for individual Bactrocera tau strains randomly selected from the F0 to F3 generations for each complex I (CI) gene strain after knockout. [file INS-33-147-s001.pdf]

Fig. S3 Chromatograms for individual *B. tau* strains randomly selected from the F0 to F3 generations for each CI gene strain after knockout.

Gene	Generations	Chromatograms
Ndufa7	F0	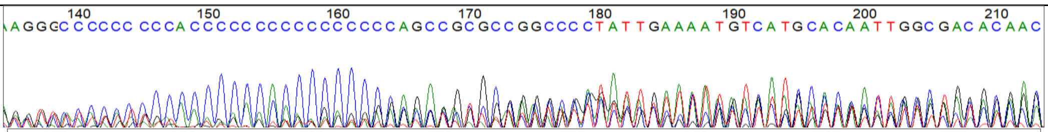
	F1	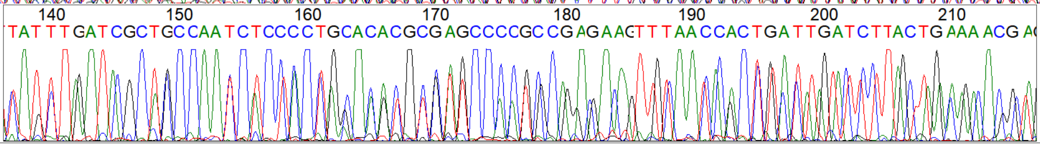
	F2	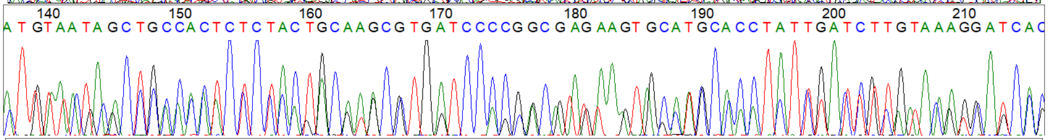
	F3	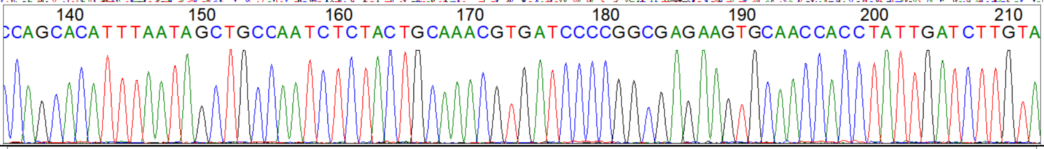
Ndufs1	F0	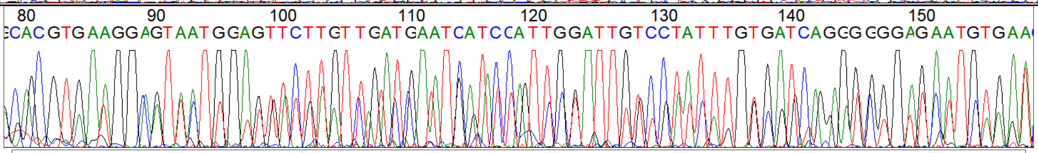
	F1	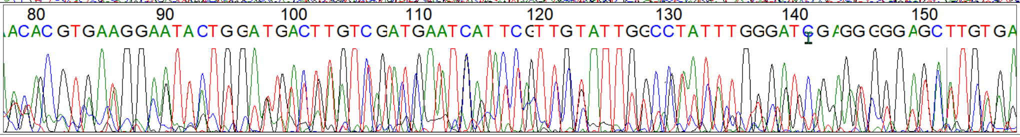
	F2	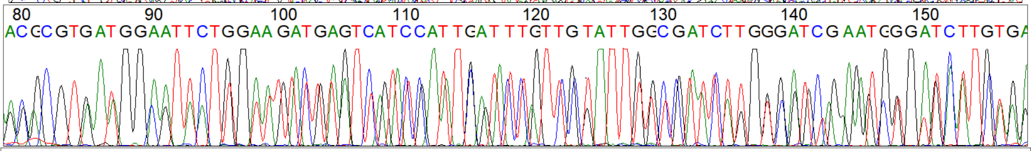
	F3	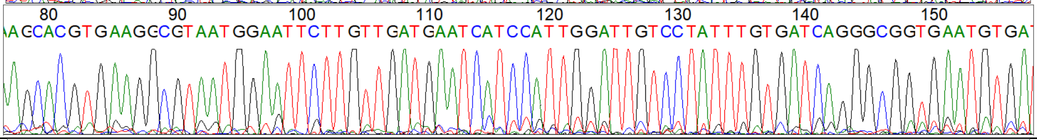
Ndufs3	F0	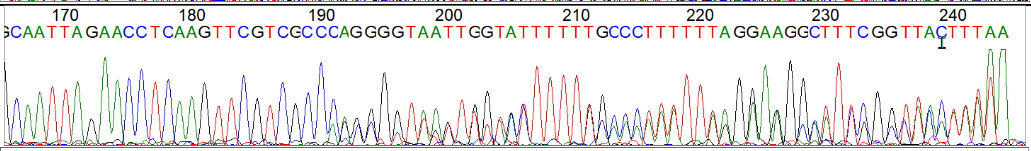
	F1	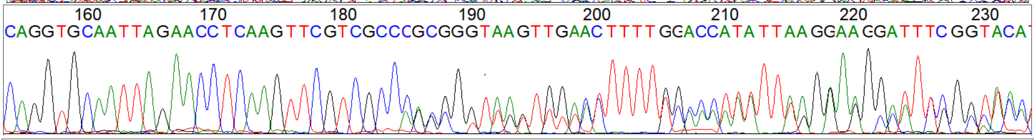
	F2	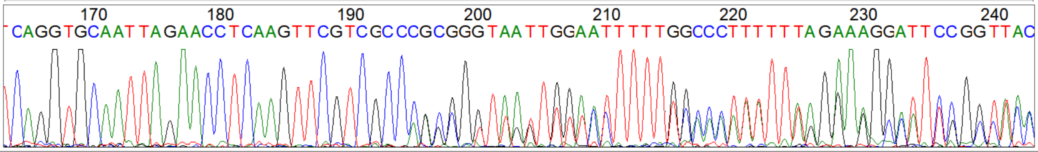
	F3	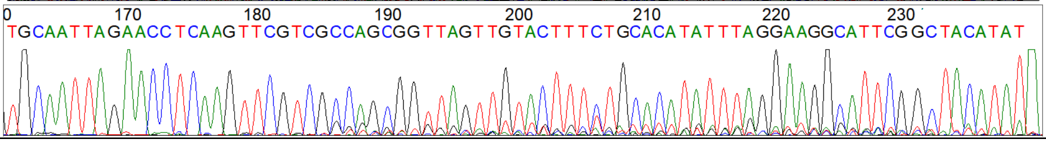
